# Utilization of intra uterine contraceptive device and associated factors among reproductive age group of family planning users in Han Health Center, Bahir Dar, North West Amhara, Ethiopia, 2018

**DOI:** 10.1186/s13104-018-4032-z

**Published:** 2018-12-22

**Authors:** Simachew Animen, Selamawit Lake, Esubalew Mekuriaw

**Affiliations:** Department of Midwifery, College of Medicine and Health Sciences, Bahir Dar University, Bahir Dar, Ethiopia

**Keywords:** IUCD, Utilization, Factors, Bahir Dar city, Ethiopia

## Abstract

**Objective:**

Aim was to assess utilization of IUCD and factors among family planning users in Han health center, Bahir Dar, Ethiopia, 2018. Two hundred forty-one participants were selected by Systematic sampling technique from June 10 to July 10, 2018. Logistic regression employed to assess association between variables with 95% CI and p value less than 0.05 was set association.

**Results:**

32 (13.3%) used intrauterine contraceptive device. Age 35–49 [AOR = 5.38, 95% CI 1.02, − 28.49] women who could read and write [AOR = 4.64, 95% CI 1.45–14.87], who were primary [AOR = 8.08, 95% CI 2.19–29.76], who were secondary [AOR = 8.89, 95% CI 1.63–48.42] who were attended college and above [AOR = 21.24, 95% CI = 5.05–89.39] and who were counseled IUCD [AOR = 3.08, 95% CI 1.26–7.54] were significant factors. Therefore, to scale up the utilization of IUCD, counseling IUCD and expanding female education should be undertaken.

## Introduction

Intrauterine contraceptive devices (IUCDs) are devices made of plastic that are inserted into women’s uterus to prevent unwanted pregnancy. They are normally small T- shaped in nature. IUCDs have been used since the beginning of the twentieth century, but became a popular contraceptive method from 1960s till now [1].

Unlike the contraceptive methods like the pill or condoms, IUCD doesn’t need to think about taking every day or using every time they have sex [2].

When compared with the use of other methods, use of IUCD results in fewer unintended pregnancies and fewer clinic visits [3].

However, the use of IUCD is still very low in Sub-Saharan Africa (SSA), including Ethiopia where the level of fertility and unmet need for family planning is high [4].

Though as shown in many countries unintended pregnancies are high, wide spread use of IUCD could prevent very high numbers of unintended pregnancies and abortions. The reproductive health impacts of unintended pregnancy and unsafe abortion are high among women’s especially in developing countries including Ethiopia. So, preventing unintended pregnancy is the first motive for improving family planning services for the reduction of maternal morbidity and mortality in Ethiopia. Understanding the factors affecting the usage of IUCD may be instructive in the design of interventions to improve family planning out comes [5, 6].

The results from this study will be used as baseline information for researchers to conduct other related findings on IUCD uses. It will also help to maximize health professionals’ effort in improving IUCD service utilizations and policy makers to redesign the existed program to wards planning services to the factor affecting utilizations of Intra Uterine Contraceptive Device among women of reproductive age group.

## Main text

### Methods

#### Study setting and period

Institutional based cross sectional study was conducted from June 10 to July 10/2018. It was conducted in Han health center in Bahir Dar city, capital of Amhara region which is located 565 km far from Addis Ababa, capital of Ethiopia. The health center has 3 health posts and 29 health professionals and 21 administrative staff, total of 50 staffs. The health center is serving for a total of 55,920 people from the surrounding areas; among these 13,216 are reproductive age women.

### Sample size and sampling techniques

The sample size was determined by using the single population formula with the assumption of 95% CI, and 18.7% intra uterine contraceptive coverage in Addis Ababa five health centers [7] with 10% non- response rate and 5% margin of error were used to obtain a total sample size of 241. Han health center was selected by simple random method. Then, study participants selected by systematic sampling technique. Minors (< 16) were included in the patient sample in my studies. According to the data from Han Health Center, the total number of women that used modern family planning methods in the last similar month with our data collection period was 450. The sampling fraction (kth) value was determined by dividing the total number of women that used family planning in the last similar month prior to our data collection period was 450. The fraction was calculated as 450**/**241 = 2 and the 1st respondent was selected by lottery method.

#### Measurement

Data were collected through face to face interview using a structured and pre-tested questionnaire. Training was given for data collectors and supervisors for 3 days on methods of extracting the information through interviewing, how to fill the information on a structured questionnaire and the ways of approaching to the respondents. Filled questioners were cheeked daily by supervisors for completeness, legibility, and consistency.

#### Statistical analysis

Data entry and cleaning were done using EPI-INFO version 7. Then data were exported to SPSS version 21 for analysis. Descriptive statistics computed and presented using table. The main outcome variable, utilization of IUCD, is binary in nature and it is labeled as “1” when the clients used IUCD, otherwise “0”. Frequencies and proportions were computed for description in relation to socio- demographic and other variables. Strength of statistical associations was determined using crude and adjusted odd ratios with 95% confidence intervals in logistic regressions to assess the association between the different predictor variables. First bivariate relationships between each independent variable and outcome were investigated using binary logistic regression model. Those variables with P- value less than 0.2 at the bivariate level were included in a multivariate logistic regression model for controlling potential confounding variables.

### Results

#### Socio-demographic characteristics

A total of 241 reproductive age women were interviewed making 100% response rate. Majority, 169 (70.1%) of them were in the age group of 25–34 years. Regarding to the marital status of the participants, majority 208 (86.3%) of them were married and a few respondents 2 (0.8%) were widowed (Table 1).

**Table 1:** Distribution of study participants by their Scio- demographic characteristics in Han Health Center, Bahir Dar, Ethiopia, 2018 (n = 241)

	Frequency	Percent
Age of the women		
15–24	37	15.4
25–34	169	70.1
35–49	35	14.5
Ethnicity		
Amhara	232	96.3
Others	9	3.7
Religion		
Orthodox	202	83.8
Muslim	36	14.9
Protestant	3	1.3
Marital status		
Married	208	86.3
Single	24	10
Divorced	7	2.9
Windowed	2	0.8
Education of the women		
Unable to read and write	16	6.6
Able to read and write	27	11.2
Primary [1–3, 10–14]	124	51.6
Secondary [5, 6, 15, 16]	46	19
College and above	28	11.6
Education of the husband		
Unable to read and write	8	3.8
Able to read and write	24	11.5
Primary [1–3, 10–14]	41	19.7
Secondary [5, 6, 15, 16]	72	34.6
Collage and above	63	30.4
Distance from nearest health center (km)		
< 3	199	82.6
> 3	42	17.4
Occupation		
Housewife	103	42.7
Governmental employee	39	16.3
Merchants	52	21.5
Student	11	4.6
Farming	36	14.9
Household income		
< 600	81	33.6
600–3000	107	44.4
> 3000	53	22

Ethnicity, others Tigre (n = 6) and Agew (n = 3)

#### Reproductive characteristics

Almost all 227, (94.2%) participants wanted to space between two children at 1–3 years intervals. Almost two- third of the respondents, 156 (64.7%) has not ever been counseled about IUCD.

#### Utilization of IUCD

**Fig. 1:**
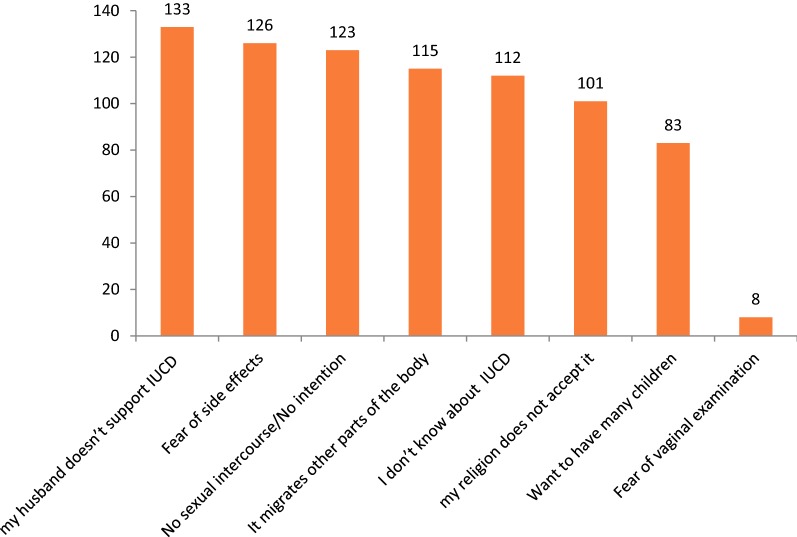
Major Reasons not to use IUCD for reproductive age group women among who have never used in Han Health Center, Bahir Dar, Ethiopia, 2018 (n = 209)

Only a few participants 32 (13.3%) had been using IUCD. The most reason mentioned by participants not to use IUCD was because of their couple doesn’t support IUCD (55.2%) followed by fear of side effects (52.3%) (Fig. 1).

#### Factors associated with utilization of IUCD

Multivariate logistic regression showed: age group 35–49, educational status of the women, and ever counseled about IUCD were independently associated with utilization of IUCD.

This study found that those mothers who were in the age group of 35–49 years were 5.38 times more likely to use IUCD than those whose age were in the age group 15–24 [AOR = 5.38, 95% CI 1.02− 28.49].

Those participants who can read and write were 4.64 times higher, those who were primary school 8.08 times higher, those who were secondary school 8.89 times higher and those who were attended college and above were 21.24 higher to use IUCD as compared to women who couldn’t read and write [AOR = 4.64, 95% CI 1.45–14.87, AOR = 8.08, 95% CI 2.19–29.76, AOR = 8.89, 95% CI 1.63–48.42 and AOR = 21.24, 95% CI = 5.05–89.39] respectively.

Those women who were counseled about IUCD were 3.08 times higher to use IUCD as compared to those who were not counseled about IUCD [AOR = 3.08, 95% CI 1.26–7.54] (Table 2).

**Table 2:** Both Bi- variate and multi-variable analysis of factors associated with utilization of IUCD in Han Health Center, Bahir Dar, Ethiopia, 2018 (n = 241)

Variable	Utilization of IUCD		COR (95% CI)	AOR (95% CI)	P value
	Yes	No			
Age					
15–24	3	34	1	1	
25–34	18	151	1.35 (0.38, 4.85)	1.54 (0.36–6.65)	0.559
35–49	11	24	5.19 (1.31, 20.64)	5.38 (1.02–28.49)	0.048
Educational status					
Unable to read and write	1	15	1		
Able to read and write	8	19	5.07 (1.66–15.56)	4.64 (1.45–14.87)	0.010
Primary school	6	118	6.52 (1.92–22.12)	8.08 (2.19–29.76)	0.002
Secondary school	8	43	7.61 (1.63–35.55)	8.89 (1.63–48.42)	0.012
Collage and above	9	19	34.25 (9.17–127.87)	21.24 (5.05–89.39)	0.000
Occupations					
Housewife	12	91	0.92 (0.25–3.46)	0.84 (0.16–4.42)	0.841
Government employee	10	29	6.88 (1.79–26.43)	2.09 (0.38–11.62)	0.395
Merchant	5	47	7.61 (1.63–35.55)	3.59 (0.63–48.42)	0.120
Student	2	9	2.44 (0.35–16.93)	4.35 (0.32–58.30)	0.268
Farmer	3	33	1	1	
Ever counseled about IUCD					
Yes	20	65	3.69 (1.70–8.01)	3.08 (1.26–7.54)	0.014
No	12	144	1		
Knowledge on IUCD					
Knowledgeable (> 6.3)	21	87	2.68 (1.23–5.84)	2.25 (0.91–5.54)	0.078
Not knowledgeable (≤ 6.3)	11	122	1		

### Discussion

The current study showed overall utilization of IUCD among reproductive age women was found to be 13.3% and was high as compared to Ethiopian Demographic and Health Survey (EDHS (2%) [8]. The difference might be brought by study area. My study area was in only one Health center, but EDHS covers many parts of Ethiopia.

However, this finding was lower than the study conducted in Addis Ababa, Ethiopia (18.7%) [7]. The most possible reason could be Addis Ababa is more developed than Bahir Dar, the awareness of the women, accessibility of IUCD might be high in Addis Ababa as compared to Bahir Dar.

This study showed that age of women was one of the significant predictor for utilization of IUCD. Women in the age group 35–49 were 5.38 times more likely to be used IUCD as compared to mothers in the age group 15–24 (AOR = 5.38, 95% CI = 1.02–28.49). This finding is consistent with a study conducted in Tigre region, Shire Endasilase town [9]. The possible explanation could be as age increases women might have many children so either they might be wanted to space births or limit the number of children. It might be also due to that as age increases they might be multipara so that they might visit health institution for antenatal care, post natal care and for delivery at the same time they might get counseling about IUCD at their visit from health provider as result they could be used IUCD.

Educational status was the other significant factor for utilization of IUCD. Those participants who could read and write were 4.64 times higher, those who were primary school 8.08 times higher, those who were secondary school 8.89 times higher and those who were attended college and above were 21.24 higher to use IUCD as compared to women who couldn’t read and write [AOR = 4.64, 95% CI 1.45–14.87, AOR = 8.08, 95% CI 2.19–29.76, AOR = 8.89, 95% CI 1.63–48.42 and AOR = 21.24, 95% CI = 5.05–89.39] respectively. This study is in line with studies done in Addis Ababa, and Tigre region, Shire Endasilase town [7, 9]. This might be due to the fact that those women who were educated might have knowledge about IUCD; they might know the negative impact of having many children in their family as well as in the country and they might have positive attitude for IUCD so that they might not accept negative misconceptions about IUCD. Therefore, these women might be used IUCD.

Counseling about IUCD was the other third significant factor for utilization of IUCD. Those participants who had got counseling about IUCD were 3.08 times more likely to be used IUCD as compared to their counterparts (AOR = 3.08, 95% CI = 1.26–7.54).

We have explored more but we couldn’t found other studies that support those Participants who had got counseling about IUCD were more likely to use IUCD as compared to had not got counseling. The predicted reasons might be that counseling is the best discussion center to address negative misconceptions about IUCD.

### Conclusions

- IUCD utilization was found to be high. Age group 35–49, educational status of women and counseling about IUCD were a significant predictor for utilization of IUCD. Therefore, to scale up more IUCD utilization, make available the service at youth friendly service area to address young women, Strengthen education level of women, and all health professionals should give counseling for all reproductive women that came for any purpose in any units about IUCD.

## Limitation of the study

- The utilization of IUCD was self-reported by the respondents and there was no other way of verifying the utilization.

## Abbreviations

BDU: Bahir Dar University
CI: confidence interval
CPR: contraceptive prevalence rate
FP: family planning
IUCDs: intra uterine contraceptive devices
MCH: Maternal and Child Health
MMR: Maternal Mortality Ratio
NGO: Nongovernmental Organization
RH: Reproductive Health
TFR: Total Fertility Rate
